# *Echinococcus granulosus*: The establishment of the metacestode in the liver is associated with control of the CD4^+^ T-cell-mediated immune response in patients with cystic echinococcosis and a mouse model

**DOI:** 10.3389/fcimb.2022.983119

**Published:** 2022-08-15

**Authors:** Xinling Hou, Yang Shi, Xuejiao Kang, Zibigu· Rousu, Dewei Li, Maolin Wang, Abidan· Ainiwaer, Xuran Zheng, MingKun Wang, Bahejiang· Jiensihan, Liang Li, Jing Li, Hui Wang, Chuanshan Zhang

**Affiliations:** ^1^ State Key Laboratory of Pathogenesis, Prevention and Treatment of High Incidence Diseases in Central Asia, Clinical Medicine Institute, The First Affiliated Hospital of Xinjiang Medical University, Urumqi, China; ^2^ Basic Medical College, Xinjiang Medical University, Urumqi, China; ^3^ Xinjiang Key Laboratory of Echinococcosis, Clinical Medicine Institute, The First Affiliated Hospital of Xinjiang Medical University, and World Health Organization Collaborating Centre on Prevention and Case Management of Echinococcosis, Urumqi, China; ^4^ Department of Hepatic Hydatid and Hepatobiliary Surgery, Digestive and Vascular Surgery Centre, The First Affiliated Hospital of Xinjiang Medical University, Urumqi, China

**Keywords:** cystic echinococcosis, protoscoleces, early immune response, establishment, liver, CD4^+^ T cells

## Abstract

The larval stage of the tapeworm *Echinococcus granulosus sensu lato* (*E. granulosus s.l.*) caused a chronic infection, known as cystic echinococcosis (CE), which is a worldwide public health problem. The human secondary CE is caused by the dissemination of protoscoleces (PSCs) when fertile cysts are accidentally ruptured, followed by development of PSCs into new metacestodes. The local immune mechanisms responsible for the establishment and established phases after infection with *E. granulosus s.l*. are not clear. Here, we showed that T cells were involved in the formation of the immune environment in the liver in CE patients and *Echinococcus granulosus sensu strict* (*E. granulosus s.s.*)*-*infected mice, with CD4^+^ T cells being the dominant immune cells; this process was closely associated with cyst viability and establishment. Local T2-type responses in the liver were permissive for early infection establishment by *E. granulosus s.s.* between 4 and 6 weeks in the experimental model. CD4^+^ T-cell deficiency promoted PSC development into cysts in the liver in *E. granulosus s.s.*-infected mice. In addition, CD4^+^ T-cell-mediated cellular immune responses and IL-10-producing CD8^+^ T cells play a critical role in the establishment phase of secondary *E. granulosus s.s.* PSC infection. These data contribute to the understanding of local immune responses to CE and the design of new therapies by restoring effective immune responses and blocking evasion mechanisms during the establishment phase of infection.

## Introduction

Cystic echinococcosis (CE), caused by the larval stage of the cestode parasite *Echinococcus granulosus sensu lato* (*E. granulosus s.l.*), is one of the most deleterious heminthic diseases, which remains a public health and socioeconomic concern of increasing importance in the world (Wen et al., 2019). Although various organs of CE patients may be invaded by the oncosphere, approximately 70% of patients are seen in the liver and approximately 20% are seen in the lungs (Wen et al., 2019). According to the ultrasound image and morphological changes in the structure of hepatic cystic, hepatic CE has been classified into five categories (CE1 to CE5) and three stages: the biologically active stage (CE1 and CE2), transitional stage (CE3a, b), and biologically inactive stage (CE4 and CE5) (WHO Informal Working Group, 2003). In accordance with cyst status, CE1, CE2 and CE3a are early stages, while CE4 and CE5 are late stages (Li et al., 2020). However, the local immune response in the early stages of hydatid cyst establishment in hepatic CE is poorly understood.

Secondary CE can be divided into two stages in a mouse model: an early stage in which the infection is established by days 20–30 [protoscoleces (PSCs) develop into hydatid cysts], followed by a late stage in which previously differentiated cysts grow and eventually become fertile cysts (Richards et al., 1983). Recently, some reports have also shown that strong local control of inflammation modulates the establishment of infection during the initial phase of PSC differentiation into hydatid cysts; in addition, early and local Th2-type responses are associated with immune responses within the peritoneal cavity of mouse model (Mourglia-Ettlin et al., 2011). There is scarce information available concerning the local immune responses and their course in the liver, especially regarding establishment-phase immunity, during the early stages of *E. granulosus s.l.* infection in experimental models.

There is significant activation of cell-mediated immunity, including cellular inflammatory responses and pathological changes in the early stages of CE (Riley et al., 1984; Riley et al., 1985; Riley and Dixon, 1987; Zhang et al., 2012). The vulnerability of *E. granulosuss.l.* PSCs to immune effectors is thought to be more prominent amid the early stages of infection, with only a little rate of parasites being able to develop into hydatid cysts (Liu et al., 1992; Dematteis et al., 2003). Previous studies have shown that T-cell-mediated responses play a key role in the immunology of hydatid cysts in both the mouse experimental model and CE patients (Rogan, 1998; Yasen et al., 2021b). Accumulated evidence has indicated that T lymphocytes are the predominant inflammatory cells at the site of local host responses to hydatid cysts in humans, cattle and sheep infected with CE (Sakamoto and Cabrera, 2003; Vatankhah et al., 2015; Vismarra et al., 2015; Jafari et al., 2019). In our prior work, we also found that T cells were the most frequent infiltrating cells surrounding the hepatic CE lesions during the establishment phase in experimental mice (after 2 weeks) (Li et al., 2019). Zhang et al. also reported that Th1-type immune response is stimulated by early, establishment phase cysts, while Th2, immunosuppressive-type profile are associated with established phase cysts (Zhang et al., 2003).

In this study, we first evaluated histopathological changes and the formation of the immune microenvironment and then identified the infiltration of T cells around the pericyst in the liver of CE patients with cysts of different stages. Furthermore, we confirmed the critical time window of PSC development into the cystic form and analyzed the T-cell phenotype and associated secreted cytokines in the liver of the mouse model after infection. In addition, our study demonstrated that CD4 T-cell deficiency could favor the development from PSCs to hydatid cysts during the establishment phase, in addition to the growth of cysts during the established phase.

## Materials and methods

### Patients and sample preparation

107 CE patients undergoing liver resection were enrolled in this study. All CE diagnosis was confirmed *via* liver biopsy. One specimen was collected close to the parasitic lesion, including the hydatid cyst (CLT, ‘close liver tissue’), and one was collected in the macroscopically normal liver distant from the lesion (DLT, ‘distant liver tissue’, at least 2 cm from the lesion), as reported by our previous publication (Zhang et al., 2020). According to the WHO-IWGE classification system, hepatic CE staging was performed by specialists from the ultrasound department (WHO Informal Working Group, 2003). The number of liver tissue samples, types of measurements (described in detail in the Supporting Information), and purpose of comparisons are included in **Table 1**. This study obtained licence from the ethics committee of the First Affiliated Hospital of Xinjiang Medical University (No. S20130418-3), and all patients provided informed written consent. All the data from CE patients are summarized in online **Supporting Table S1**.

**Table 1:** Liver samples from patients with CE used for immunological studies.

					
Experiment		Stages of patients	Number of CLT Samples	Number of DLT Samples	Number of paired CLT and DLT Samples	Comparison Between Groups
H&E			107	54	54	CLT vs. DLT
		CE1	20			CLT
		CE2	58			CLT
		CE3	9			CLT
		CE4	20			CLT
Masson			103	48	48	CLT vs. DLT
		CE1	20			CLT
		CE2	58			CLT
		CE3	8			CLT
		CE4	17			CLT
IHC	CD4		89	43	43	CLT vs. DLT
	CD8		89	43	43	CLT vs. DLT
	CD4	CE1	20			CLT
		CE2	43			CLT
		CE3	8			CLT
		CE4	18			CLT
	CD8	CE1	20			CLT
		CE2	43			CLT
		CE3	8			CLT
		CE4	18			CLT

### *E. granulosus s.s.*-infected mouse model

Wild-type (WT) female C57BL/6 (B6) mice were purchased from Beijing Vital River Experimental Animal Technology Co., Ltd. CD4 knockout (KO) mice were kindly provided by Dr. Zhexiong Lian (South China University of Technology, Guangzhou, China). According to the guidelines for experimental animals, all mice were maintained under specific pathogen-free conditions. Study protocol was approved by the Animal Ethical Committee of the First Affiliated Hospital of Xinjiang Medical University (No. 20140411-05).

*E. granulosus s.s.* PSCs were isolated from hydatid cysts with the sheep origin, and then washed ten times in phosphate-buffered saline, containing 1000 mg/mL penicillin and 1000 U/mL streptomycin (HyClone, Beijing, China) (Li et al., 2020). The vitality of *E. granulosus s.s.* PSC was confirmed to be more than 95% by 1% methylene blue exclusion, and were used in mice. Mice were inoculated *via* the hepatic portal vein with live PSCs in saline as previously described (Li et al., 2019), whereas control mice were injected with isotonic saline.

### Isolation of mononuclear cells from mouse liver and spleen tissue

A single-cell suspension of mononuclear cells was isolated from the liver and spleen as described previously (Ma et al., 2017). Livers were homogenized, and mononuclear cells were isolated by gradient centrifugation with 40% Percoll (GE Healthcare, Pittsburgh, PA). Spleens were disrupted between two glass slides, passed through a 74 μm nylon mesh, lysed with red blood lysis buffer and washed with PBS containing 0.2% BSA (Biolegend, San Diego, CA). The cells were then counted manually on a hemocytometer.

### Flow cytometry

A single-cell suspension of mononuclear cells from mouse samples was incubated in PBS buffer (PBS containing 0.2% BSA and 0.1% sodium azide) in the presence of neutralizing monoclonal antibodies against CD16/CD32 (Fc Block; Biolegend, San Diego, CA) for 20 min at 4°C prior to staining. For cell surface flow cytometry, 0.5×10^6^ cells per tube were stained with a mixture of fluorochrome-conjugated monoclonal antibodies, including NK1.1(PK136), CD3 (17A2), CD4 (GK1.5), CD8 (53-6.7), CD69 (H1.2F3), CD44 (IM7), CD62L (MEL-14) (Biolegend, San Diego, CA), in PBS for 30 min at 4°C in the dark.

For intracellular flow cytometry, 1×10^6^ cells per tube were cultured in the presence of Cell Stimulation Cocktail (Thermo Fisher, eBioscience, MA) according to the manufacturer’s instructions and incubated at 37°C for 4 hrs. Cells were stained for surface markers using antibodies (NK1.1(PK136), CD3 (17A2), CD4 (GK1.5), CD8 (53-6.7)) for 30 min at 4°C, and then fixed, permeabilized, and stained with the intracellular antibodies, including granzyme B (GB11), IFN-γ (XMG1.2), IL-4 (11B11), IL-17A (TC11-18H10.1), IL-10 (JES5-16E3) (Biolegend, San Diego, CA). Flow cytometer was performed using an LSRFortessa (BD Biosciences, San Diego, CA), and data were analyzed with FlowJo software 7.6.1 (Treestar, San Carlos, CA).

### Immunohistochemistry

For immunohistochemistry analysis, liver sections were de-paraffinized in two changes of xylene for 15 minutes each and then hydrated through a graded series of ethanol (100, 95, 70, 50%) and deionized water, each for 5 minutes. The tissue sections were processed for heat-mediated antigen-retrieval using Tris-EDTA or citric acid buffer for 15 minutes (ZSGB-BIO, Beijing), and then cooled at room temperature. Sections were blocked for 1 hr at room temperature in PBS with 10% goat serum (blocking buffer), and then incubated at 4°C overnight with primary antibodies in blocking buffer (anti-human CD4, 1:500; anti-human CD8, 1:1000; anti-mouse CD4, 1:1000; anti-mouse CD8, 1:500; anti-mouse α-Sma, 1:1000, Abcam, Cambridge, UK). The next day, sections were washed three times with PBS for 5 min, and incubated for 2 hrs at room temperature with secondary antibody (goat anti-rabbit F(ab’)2-HRP). Staining was developed using 3, 3’ Diaminobenzidine (DAB) substrate kit (Abcam) according to the manufacturer’s instructions. Staining was then assessed at × 100 or × 200 magnification in a total of 3-5 fields/section/sample, using cellSens Dimension software (Olympus) for computerized quantification and results were expressed as the intensity of positive staining per field.

### Statistical analysis

The data were analyzed using GraphPad Prism 6.0 (GraphPad Software, San Diego, CA). The results are present as the mean ± standard deviation. Differences between only two groups were statistically analyzed by Student’s *t* test (parametric) or the Mann–Whitney U test (nonparametric). Differences between more than two groups were statistically analyzed by one-way ANOVA. A *P* value < 0.05 was considered to indicate a significant difference for all experiments. (*P value<0.05; **P value<0.01; ***P value<0.001; ****P value<0.0001).

## Results

### Clinical characteristics, histopathology and immune microenvironment of the liver in CE patients at different stages

Overall, 107 CE patients were included during the study period., One cyst and multiple cysts were observed in 84 (78.5%) and 23 (21.5%) of the patients, respectively. The diameter of the cysts ranged from 1.1 to 17.9 cm, with a mean size of 8.1 ± 3.3 cm. In 66 (61.7%) cases, cysts were seen in the right lobe of the liver; in 31 cases (29.0%), the left lobe was infected; in 4 cases (3.7%), the middle lobe was infected; and in 5 cases (4.8%), both the right and left lobes were involved. With regard to the stages of CE cysts, 20 (18.7%) patients were classified as CE1, 58 (54.2%) patients were classified as CE2, 9 (8.4%) patients were classified as CE3, and 20 (18.7%) patients were classified as CE4 (**Table S1**).

**Figure 1:**
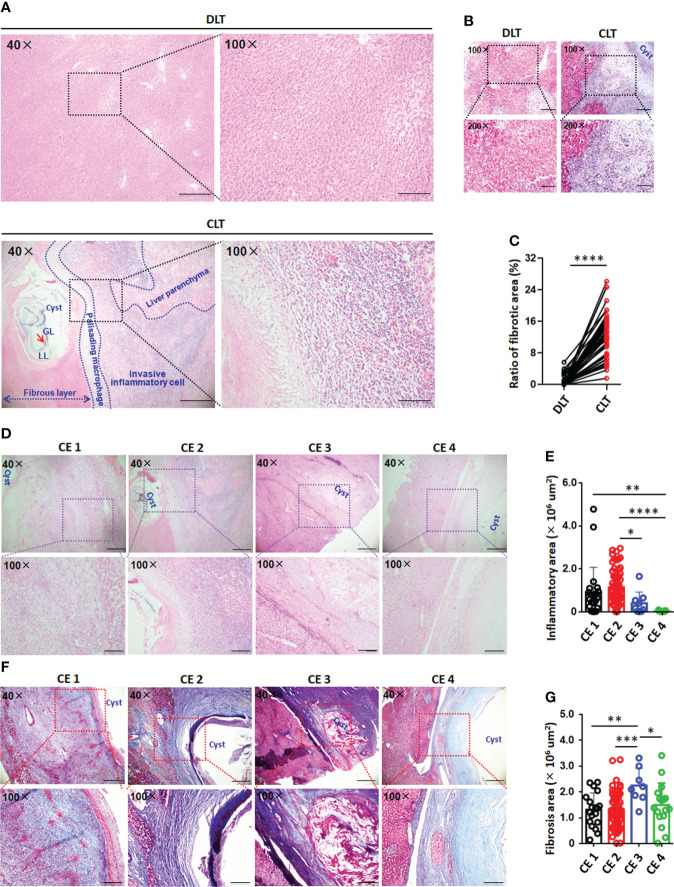
Histopathology and immune microenvironment of the liver in CE patients with hydatid cysts. **(A)** Representative hematoxylin and eosin (H&E) staining (left panel 40×, enlarged 100× on the right panel) of paired liver section from CE patients. Close liver tissue showed the cysts, fibrous layer, invasive inflammatory cell regions and liver parenchyma, delimited with the dotted line. **(B)** Representative liver fibrosis (upper panel 100×, enlarged 200× on the lower panel) as determined by Masson staining on paired liver tissue samples close to lesion (CLT) versus those distant from lesion (DLT) from CE patients. **(C)** The fibrosis area of liver section was quantified using cellSens Diemension software, the ratio of fibrosis area and total area (%) was counted from **(B)** (n=48). **(D)** Representative inflammatory cell infiltration (upper panel 40×, enlarged 100× on the lower panel) in liver section from CE patients at different stages. **(E)** The inflammatory cell infiltration area of liver section was quantified using cellSens Diemension software, the inflammatory area size (μm^2^) was counted from **(D)** (n=107). **(F)** Representative liver fibrosis (upper panel 40×, enlarged 100× on the lower panel) in liver section from CE patients at different stages. **(G)** The fibrosis area of liver section was quantified using cellSens Diemension software, the fibrosis area size (μm^2^) was counted from **(F)** (n=103). Data are expressed as mean ± SD, analyzed by Student’s t tests. *P < 0.05, **P < 0.01, ***P < 0.001, ****P < 0.0001.

Histological analysis showed the formation of the immune microenvironment around the pericyst in the liver of CE patients; its composition, from the inside to the outside, mainly included hydatid cysts, fibrous layers, palisading monocyte and/or macrophage areas, infiltrating lymphocyte areas and normal liver parenchyma (**Figure 1A**). In addition, the size of the inflammatory area surrounding the cysts in the CLT was significantly increased in CE1 and CE2 cysts compared with CE3 and CE4 cysts. Liver tissue damage and fibrosis were observed in the periparasitic areas in CE patients (**Figures 1D** **, E**). By Masson staining, the quantity of collagen fibers and fibrosis was significantly higher in CLT than in DLT from paired liver tissue samples. Moreover, collagen deposition accumulated around the cysts in CLT was significantly increased in patients with CE3 cysts compared with CE1, CE2 and CE4 cysts (**Figures 1B, C, F, G**).

Liver function analysis revealed that the levels of serum alanine aminotransferase (ALT, mean: 58.8 U/L) and aspartate aminotransferase (AST, mean: 46.9 U/L) in CE patients were higher than the normal range, while the alkaline phosphatase (ALP) level (mean: 100.4 U/L) did not change significantly (Mirzavand et al., 2020) (**Table S1**). Furthermore, the levels of ALT, AST and ALP were higher in patients with CE1 and CE2 cysts than in patients with CE3 and CE4 cysts, and ALT and ALP levels were significantly different between patients with CE1 and CE3 cysts and between patients with CE2 and CE4 cysts, respectively (**sFigure 1**).

### T cells accumulated in the periparasitic areas of the liver in CE patients at different stages

**Figure 2:**
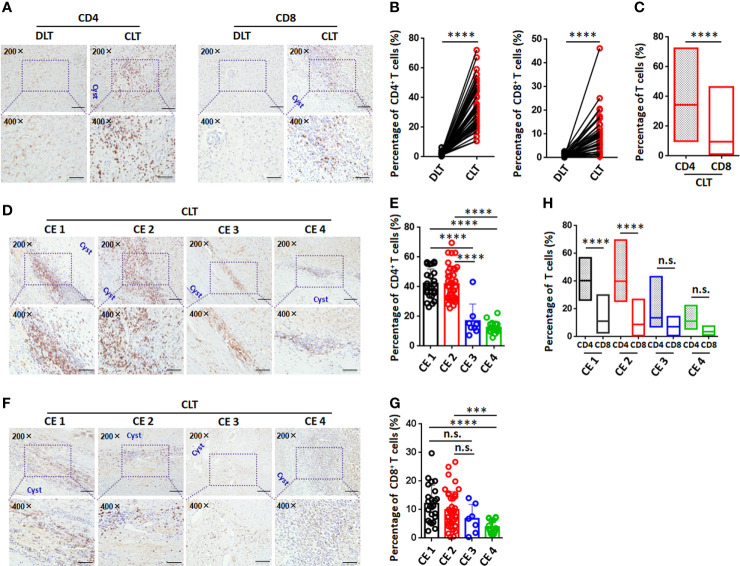
Immunohistochemical analysis of CD4^+^ T cells and CD8^+^ T cells in the liver in CE patients at different stages. **(A)** Representative images for CD4 and CD8 staining (upper panel 200×, enlarged 400× on the lower panel) on paired liver sections from CE patients. **(B)** Percentage of infiltrating CD4^+^ T cells or CD8^+^ T cells in paired liver tissue, from **(A)** (n=43). **(C)** Comparison of CD4^+^ T cells to CD8^+^ T cells percentage on CLT from CE patients. **(D)** Representative images for CD4 staining (upper panel 200×, enlarged 400× on the lower panel) on CLT from CE patients at different stages. **(E)** The percentage of infiltrating CD4^+^ T cells on CLT, from **(D)** (n=89). **(F)** Representative images for CD8 staining (upper panel 200×, enlarged 400× on the lower panel) on CLT from CE patients at different stages. **(G)** The percentage of infiltrating CD8^+^ T cells on CLT, from **(F)** (n=89). **(H)** Comparison of CD4^+^ T cells to CD8^+^ T cells percentage on CLT from CE patients at different stages. Data are expressed as mean ± SD, analyzed by Student’s t tests or one way ANOVA. ***P < 0.001, ****P < 0.0001, no significance(n.s.) *P* > 0.05.

To elucidate the pericyst T-cell distribution in the liver, 89 samples from 107 CE patients were studied. The numbers of both CD4^+^ T cells and CD8^+^ T cells were greatly increased in the periparasitic areas of CLT specimens compared to DLT specimens (**Figures 2A, B**). In addition, the increase in the number of CD4^+^ T cells was larger than that of CD8^+^ T cells in the pericyst areas in the CLT specimens from CE patients (33.7% for CD4^+^ T cells vs. 11.5% for CD8^+^ T cells) (**Figure 2C**), suggesting that a predominantly CD4^+^ T-cell-mediated immune response was critical in the liver of persistent CE patients.

Furthermore, we analyzed T-cell infiltration around liver hydatid cysts in CE patients at different stages of disease. The percentage of CD4^+^ T cells was significantly higher in the pericyst area in the CLT specimens from CE1 and CE2 patients compared to CE3 or CE4 patients (**Figures 2D** **, E**). The percentage of CD8^+^ T cells was significantly higher in the pericyst area in the CLT specimens from CE1 and CE2 patients when compared to CE4 patients. However, there was no significant difference between CE1 and CE2 patients or between CE3 and CE4 patients (**Figures 2F** **, G**). Further analysis showed that the proportion of CD4^+^ T cells was significantly greater than that of CD8^+^ T cells in the pericyst area in CE1 and CE2 patients. As the disease progressed to transitional CE3 or inactive CE4, there was no significant difference in the proportions of CD4^+^ T cells and CD8^+^ T cells in the pericyst area in the livers of patients (**Figure 2H**). These results suggested that the predominant recruitment of CD4^+^ T cells to the pericyst area was closely related to active lesions (CE1 and CE2).

### T cells accumulated in the periparasitic areas of the liver in the mouse model during *E. granulosus s.s.* establishment

**Figure 3:**
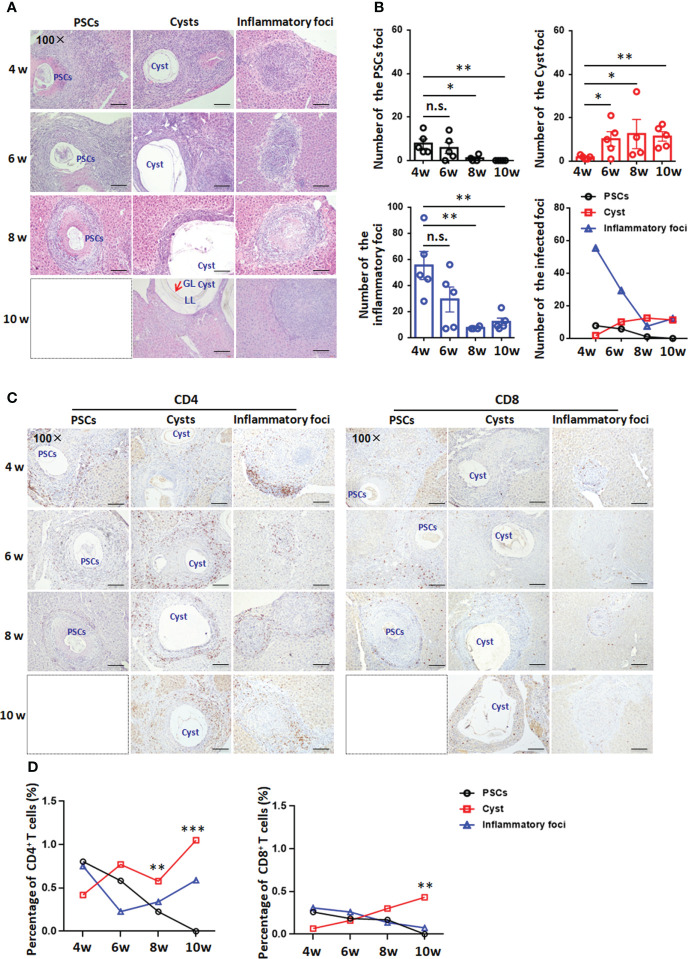
Histopathology and immunohistochemical analysis of CD4^+^ T cells and CD8^+^ T cells in the liver in a mouse model during *Egranulosus s.s.* establishment. **(A)** Histopathological alterations of the liver from mice model during *Egranulosus s.s.* establishment (protoscoleces develop into hydatid cysts). H&E staining of liver sections (original magnification 100×). **(B)** Hepatic granulomatous response to *Egranulosus* infection establishment. Liver histological reaction at each infectious foci was scored as (1) PSCs foci, visible PSCs, only composed of infiltrating inflammatory cells and fibrosis; (2) infectious foci, cystic structure composed of the germinal layer and laminated layer, as well as fibrosis (adventitial layer, AL); (3) inflammatory foci, parasite-free, composed of macrophages, lymphocytes, and other inflammatory cells. **(C)** Representative images for CD4 and CD8 staining on liver sections (original magnification 100×) from mouse model during *Egranulosus s.s.* establishment. **(D)** The percentage of infiltrating CD4^+^ T cells or CD8^+^ T cells on liver sections, from **(C)**. 4-5 mice per group. PSCs, protoscoleces; LL, laminated layer; GL, germinal layer; Cyst, hydatid csyst. The dotted box represents the absence of PSC-type lesions. Data are expressed as mean ± SD or mean, analyzed by Student’s t tests or one way ANOVA. *P < 0.05, **P < 0.01, ***P < 0.001, no significance(n.s.) *P* > 0.05.

In experimental mice, at 4, 6 and 8 weeks, lymphocytes infiltrates around the *E. granulosus s.s.* inoculum in all infected groups, and a few PSCs were still present in the liver lobe. In addition, visible PSCs in the liver disappeared at 10 weeks. At 8 weeks, the number of PSC foci was significantly lower than those at 4 and 6 weeks and showed a gradual decrease. Furthermore, the cysts formed in the liver were typical of the germinal layer (GL), laminated layer (LL) and adventitial layer (AL) at 4 weeks. At 6, 8 and 10 weeks, when the cysts rapidly increased in number, with more cysts than observed at 4 weeks, numerous fibroblasts and inflammatory cells were present at the periphery of the lesion(s). From 4 to 10 weeks, an obvious decrease in granuloma (inflammatory foci with fibrosis) numbers was observed (**Figures 3A** **, B**). These results indicated that between 4 and 6 weeks was a critical time window for the development of PSCs into hydatid cysts.

To verify whether the T-cell accumulation observed in CE patients was evident in the mouse model during *E. granulosus s.s.* establishment, we analyzed CD4 and CD8 expression in the liver at 4, 6, 8 and 10 weeks by IHC staining. At 4 weeks, the percentages of CD4^+^ T cells and CD8^+^ T cells were lower in the adventitia surrounding hydatid cysts than in PSC foci or inflammatory foci. At 6 weeks, the percentage of CD4^+^ T cells was significantly increased in the pericyst area, while the percentage of CD8^+^ T cells was not different among the three types of lesions. At 8 and 10 weeks, CD4^+^ T cells and CD8^+^ T cells were present in high numbers in the periparasitic area of liver hydatid cysts. In addition, the CD4^+^ T-cell population was denser than the CD8^+^ T-cell population and was scattered all around the parasitic lesions (**Figures 3C** **, D**). Therefore, local CD4^+^ T-cell predominance favored the establishment of hepatic cysts between 4 and 6 weeks.

Moreover, collagen deposition and α-Sma-positive cells were observed around PSCs, cysts and inflammatory lesions and in the portal spaces at 4, 6, 8 and 10 weeks. We found that collagen deposition and the percentage of α-Sma-positive cells gradually decreased around PSC lesions from 4 to 10 weeks, whereas they decreased and then increased around cysts and inflammatory lesions. In addition, collagen deposition and the percentage of α-Sma-positive cells were higher around cysts than around PSCs and inflammatory lesions (**sFigure 2**).

### T-cell phenotypes in the liver in the mouse model during *E. granulosus s.s.* establishment

**Figure 4:**
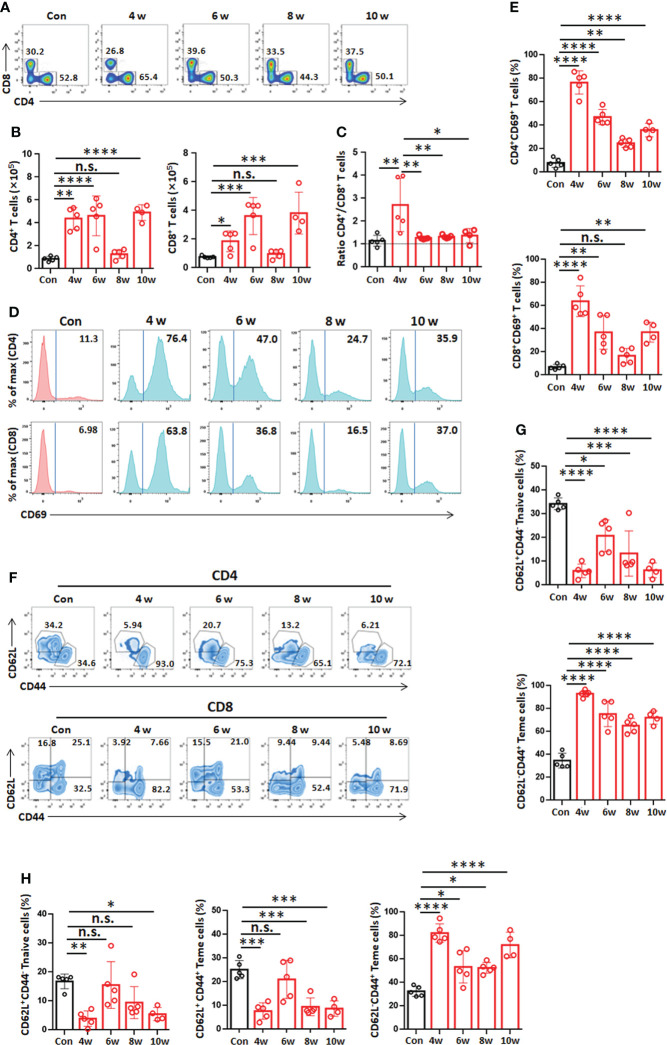
T-cell phenotypes in the liver in the mouse model during *Egranulosus s.s.* establishment. **(A)** Representative flow cytometric analysis for hepatic CD4^+^ T cells or CD8^+^ T cells from mouse model during *Egranulosus s.s.* establishment. **(B)** Absolute quantification of hepatic CD4^+^ T cells or CD8^+^ T cells from mouse model during *Egranulosus s.s.* establishment. **(C)** The ratio of CD4^+^ T cells to CD8^+^ T cells in the liver. **(D)** Representative flow cytometric analysis for hepatic CD4^+^ T cells or CD8^+^ T cells expressing CD69 from mouse model during *Egranulosus s.s.* establishment. **(E)** The percentage of hepatic CD4^+^ T cells or CD8^+^ T cells expressing CD69, from **(D)**. **(F)** Representative flow cytometric analysis for CD44 and CD62L expression in hepatic CD4^+^ T cells or CD8^+^ T cells from mouse model during *Egranulosus s.s.* establishment. **(G)** The percentages of CD4^+^ naïve T cells (Tn, CD44^-^CD62L^+^) and CD4^+^ effector memory T cells (Tem, CD44^+^CD62L^-^) on CD4^+^ T cells in the liver, from **(F)**. **(H)** The percentages of CD8^+^ naïve T cells (Tn, CD44^-^CD62L^+^), CD8^+^ central memory T cells (Tcm, CD44^+^CD62L^+^) and CD8^+^ effector memory T cells (Tem, CD44^+^CD62L^-^) in the liver, from **(F)**. 4-5 mice per group. Data are expressed as mean ± SD, analyzed by one way ANOVA. *P < 0.05, **P < 0.01, ***P < 0.001, ****P < 0.0001, no significance(n.s.) *P* > 0.05.

Hepatic T lymphocytes (NK1.1^-^CD3^+^, both CD4^+^ and CD8^+^) were analyzed by flow cytometry. These results showed a rapid increase in CD4^+^ T cells (NK1.1^-^CD3^+^) by week 4, reaching a 4-fold increase by week 6 or 10, and a slower increase in CD8^+^ T cells (NK1.1^-^CD3^+^) at week 4, reaching a 2-fold increase in the liver. However, the absolute numbers of CD4^+^ T cells and CD8^+^ T cells were not significantly different at week 8 (**Figures 4A** **, B**). The CD4^+/^CD8^+^ T-cell ratio increased significantly at week 4, and then there was no change from week 6 to 10 (**Figure 4C**), suggesting that CD4^+^ T cells might play an important role during the initial phase of PSC differentiation into hydatid cysts after infection.

Furthermore, we analyzed the activation and memory of T cells (both CD4^+^ and CD8^+^) in the liver. The percentage of CD69 expression among T cells (NK1.1^-^CD3^+^, both CD4^+^ and CD8^+^) was significantly increased from week 4 to 10 and peaked at week 4 (**Figures 4D** **, E**). At weeks 4, 6, 8 and 10, the percentage of CD4^+^ effector memory T cells (Tem, CD44^+^CD62L^-^) was higher than that of the control group, and it was significantly increased at week 4 and represented 93.04% of CD4^+^ T cells after infection (**Figures 4F** **, G**). In addition, the percentage of CD8^+^ central memory T cells (Tcm, CD44^+^CD62L^+^) was lower than that of the control group, while the percentage of CD8^+^ Tem cells was higher than that of the control group at 4, 6, 8 and 10 weeks and represented 82.20% of CD8^+^ T cells at 4 and 10 weeks (**Figures 4F** **, H**). Overall, these findings here suggest an active role for hepatic T-cell subpopulations during PSC development into cysts during *E. granulosus s.s.* infection.

### Distribution of T-cell subsets in the liver in the mouse model during *E. granulosus s.s.* establishment

**Figure 5:**
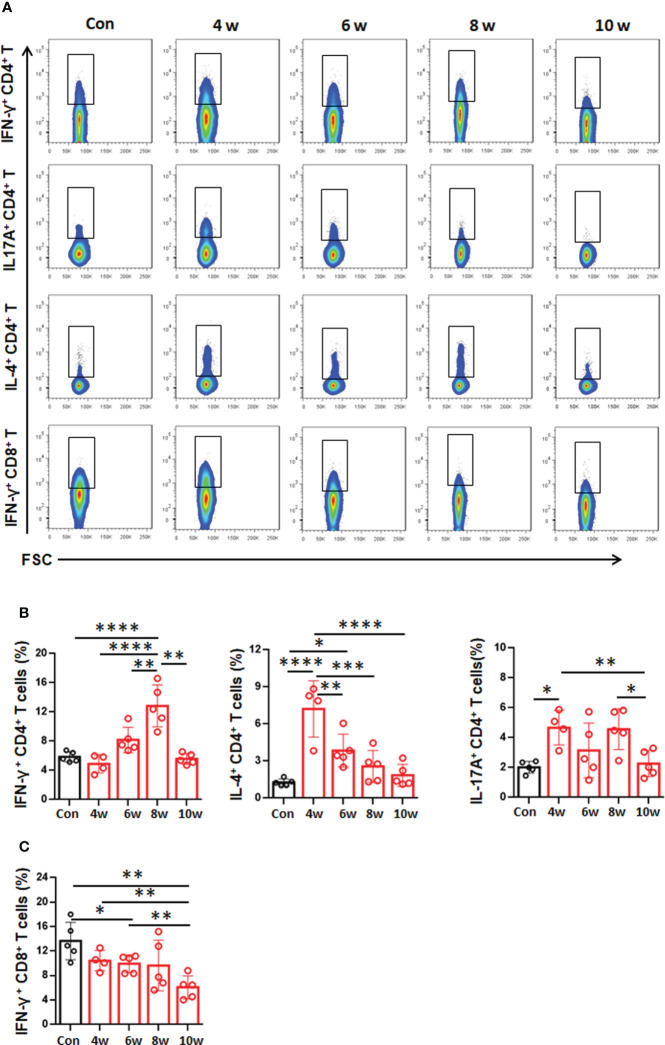
Distribution of T-cell subsets in the liver in the mouse model during *Egranulosus s.s.* establishment. **(A)** Representative flow cytometric analysis for IFN-γ, IL-17A and IL-4 production in CD4^+^ T-cell subsets or IFN-γ production in CD8^+^ T-cell subsets in the liver from mouse model during *Egranulosus s.s.* establishment. **(B)** The percentage of IFN-γ, IL-17A and IL-4 production in CD4^+^ T-cell subsets in the liver, from **(A)**. **(C)** The percentage of IFN-γ production in CD8^+^ T-cell subsets in the liver, from **(A)**. 4-5 mice per group. Data are expressed as mean ± SD, analyzed by one way ANOVA. *P < 0.05, **P < 0.01, ***P < 0.001, ****P < 0.0001, no significance(n.s.) *P* > 0.05.

The local cytokine profile in the liver during *E. granulosus s.s.* establishment has been rarely described. Hence, we analyzed T-cell subset-related cytokines in livers from infected mice. The CD4^+^IFN-γ^+^ T-cell (T1 type, NK1.1^-^CD3^+^) percentage was significantly higher at 8 weeks than at 4, 6 and 10 weeks or than in the control group (**Figures 5A** **, B**). At weeks 4 and 6, the CD4^+^IL-4^+^ T-cell (T2 type, NK1.1^-^CD3^+^) percentage was significantly increased, reaching a 5.66-fold increase at week 4, and then gradually decreased from 6-10 weeks (**Figures 5A** **, B**). At 4 and 8 weeks, the CD4^+^IL-17A^+^ T-cell (T17 type, NK1.1^-^CD3^+^) percentage was significantly higher than that at 6 and 10 weeks or that in the control group (**Figures 5A** **, B**). At 4, 6 8 and 10 weeks, the CD8^+^IFN-γ^+^ T-cell (T1 type, NK1.1^-^CD3^+^) percentage gradually decreased and was significantly lower at 6 and 10 weeks than that of the control group (**Figures 5A** **, C**). These results showed that the T2-type local immune response seems to predominate at 4 and 6 weeks, promoting PSC development to the immune-resistant cystic stage during early infection.

### CD4 T-cell deficiency promotes PSC development into cysts in the liver in *E. granulosus s.s.*-infected mice

**Figure 6:**
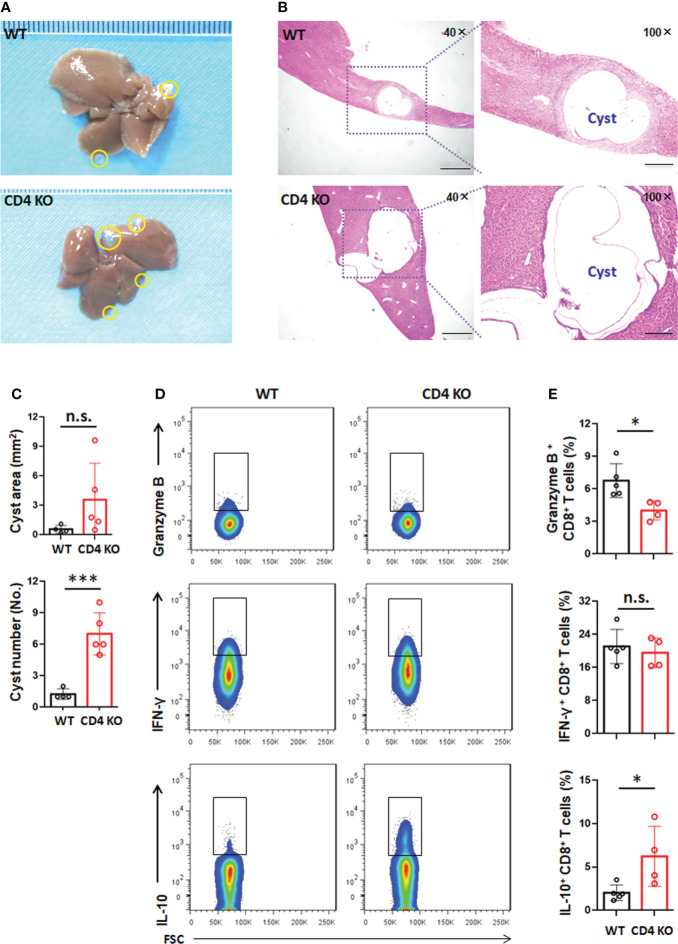
CD4 T-cell deficiency promotes PSC development into cysts in the liver in *E granulosus s.s.*-infected mice. **(A)** Representative image of livers of wild type (WT) or CD4 KO mice at week 10 post infection. The yellow line encircles metacestode tissues. **(B)** Representative hematoxylin and eosin (H&E) staining (left panel 40×, enlarged 100× on the right panel) of liver section from WT or CD4 KO mice at week 10 post infection. **(C)** Area (mm^2^) and number of the infectious foci (hydatid cyst) in the liver of WT or CD4 KO mice at week 10 post infection. **(D)** Representative flow cytometric analysis for granzyme B, IFN-γ and IL-10 production in CD8^+^ T cells in the liver from WT or CD4 KO mice at week 10 post infection. **(E)** The percentage of granzyme B, IFN-γ and IL-10 production in CD8^+^ T cells in the liver, from **(D)**. 4-5 mice per group. Data are expressed as mean ± SD, analyzed by Student’s t tests. *P < 0.05, ***P < 0.001, no significance(n.s.) *P* > 0.05.

To better understand the role of CD4 T cells in the establishment and growth of hydatid cysts, we infected wild-type (WT) or CD4 T-cell-deficient (CD4 KO) mice with *E. granulosus s.s*. We found that CD4 T-cell deficiency promoted liver hydatid cyst growth, but there was no significant difference compared with WT (*P >*0.05). The cyst number in the liver was significantly increased in CD4 T-cell-deficient mice compared to WT mice (*P <*0.001) (**Figures 6A** **–C**). Furthermore, mild collagen deposition was distributed around granulomatous inflammatory lesions and in the portal spaces. Picric acid-Sirius red and α-Sma staining, while higher in CD4 KO mice than in WT mice, was not significantly different (*P >*0.05). Collagen deposits were mainly localized around the cysts (**sFigure 3**). These results indicated that CD4 T-cell deficiency is more important for the development of PSCs into hydatid cysts during the establishment phase, in addition to promoting the growth of cysts in the established phase.

We further analyzed the effector characteristics of CD8^+^ T cells in WT and CD4 KO mice. We observed that the percentage of CD8^+^ T cells expressing granzyme B (NK1.1^-^CD3^+^CD8^+^granzyme B^+^) was significantly decreased while the percentage of CD8^+^ T cells expressing IL-10 (NK1.1^-^CD3^+^CD8^+^IL-10^+^) was significantly increased in the liver of CD4 KO mice. In addition, there was no significant difference in the expression of IFN-γ in CD8^+^ T cells (NK1.1^-^CD3^+^CD8^+^IFN-γ^+^) in the liver in CD4 KO mice (**Figures 6D** **, E**). However, a lower level of IFN-γ was present in splenic CD8^+^ T cells from CD4 KO mice. The percentage of splenic CD8^+^ T cells expressing granzyme B or IL-10 showed no difference between WT and CD4 KO mice (**sFigure 4**). These data suggested that the increased secretion of IL-10 by liver CD8^+^ T cells could facilitate the development of PSCs into hydatid cysts in the absence of CD4 T cells.

## Discussion

Hydatid cysts, the larval stage of *E. granulosus s.s.*, are able to survive in host tissues for long periods of time, and the liver is the major site of CE involvement. The chronic infection induces an immune imbalance on the hepatic tissue, and gradually forms an immune microenvironment (Jafari et al., 2019; Wen et al., 2019). The cellular distribution and functions of immune mediators in the liver are different from those in the periphery; analysis at the site of infection is thus important for deciphering anti-*E. granulosus s.s.* immunity. Local immune responses elicited in the liver, especially those observed at an early stage of infection, have seldom been studied during *E. granulosus s.s.* PSCs establishment in experimental secondary infection model. PSCs are the infective stage for the definitive host (mainly dogs), but they are also able to reverse differentiation, generating new cysts in a relatively short period of time when released in the intermediate host after cyst rupture (Breijo et al., 2008). Therefore, we focused on the characterization of the early immune response at the sites of local host-parasite responses during infection establishment, *e.g.*, the liver.

In the present work, we have shown that a variety of infiltrating inflammatory cells and fibroblasts gathered surrounding hydatid lesions in the liver of CE patients, producing an immune microenvironment between the LL of the tapeworm and normal hepatic tissue (**Figure 1A**). Histologically, immune microenvironment formation and persistent liver fibrosis may have contributed to the control of parasite overgrowth (Yasen et al., 2021a). Our results demonstrated that local immune response patterns and the specific infiltrating immune cell profiles of CE patients were altered during disease progression and varied across patients (Ahn et al., 2015). The predominance of CD4^+^ T cells compared to CD8^+^ T cells was significantly increased in the CLT, and the percentage of CD4^+^ T cells was obviously higher in CE patients in the active stages of disease (CE1 and CE2), thus suggesting that T cells, especially CD4^+^ T cells, in the lesion microenvironment play a significant regulatory role in the development and progression of CE and are closely related to CE cyst viability. Consistent with our work, previously report showed that a larger T-cell population (CD4^+^ T cells) was at the site of host tissue responses around hydatid cysts in humans and sheep infected with CE (Vatankhah et al., 2015; Vismarra et al., 2015). Ample evidence indicates that a strong Th2 response (mainly *via* CD4^+^ T-cell contributions) correlates with susceptibility to disease in the active CE1 and CE2 stages whereas a Th1 response correlates with protective immunity against the transitional CE3 and inactive CE4 and CD5 stages and that Th1 and Th2 responses coexist (Li et al., 2020).

Much of the evidence from clinical CE patients suggests that T-cell control of established infections (established phase) is dominated by CD4^+^ T cells (Jafari et al., 2019; Yasen et al., 2021b). We further determined the time points at which PSCs develop into hydatid cysts during the establishment phase and then analyzed early and local T-cell immune responses using experimental mice with intrahepatic infection induced *via* portal vein injection. Histological examination showed that viable, intact PSCs persist in the host liver tissue for 8 weeks after infection; importantly, the bulk of inoculated PSCs are eliminated during the establishment phase, i.e., between 4 and 6 weeks, during which infective forms survive early host defenses and PSCs develop into cyst-bearing laminated layers during a specific time window. Previous studies have also indicated that PSCs became cysts in a relatively short time after subcutaneous or intraperitoneal injection in a murine model (Riley et al., 1985; Mourglia-Ettlin et al., 2011). These findings confirmed that T-cell activity may be critical for the regulation of early development and growth of the parasite. In line with these studies, our findings demonstrated that the CD4^+^ T-cell number showed a faster increase than the CD8^+^ T-cell number in the lesion microenvironment, with the former being the main contributor to the total T-cell increase during the *E. granulosus s.s.* establishment phase in mouse model. In addition, the percentages of CD69 expression and Tem cells (both CD4 and CD8) were significantly increased during the establishment phase (4 weeks) and then decreased to a certain extent (from 6 to 10 weeks). These findings indicate that *E. granulosus* infection can result in a transition to T-cell immune activation and a memory response, which develop during the early stage of *E. granulosus s.s.* infection when PSCs differentiate into cysts.

Recent studies found that the Th1-type cytokine profile was predominant during the early stages after infection (3-4 weeks); then, a shift to Th2-type cytokines took place at week 4 in *E. granulosus s.s.* PSCs-infected mice (Rostami-Rad et al., 2018). It has been reported that Th1-type immune response was stimulated by early, establishment phase cysts, while Th2-type, immunosuppressive-type profile are associated with established phase cysts (Zhang et al., 2003). Here, we have shown that the T2-type cytokine IL-4 is predominant in the lesion environment during the early phase of establishment, which may play an important role in the regulation of the immune response in favor of PSC development into the immune-resistant cystic stage. As the infection progresses, a permanent T1-type response follows (high levels of IFN-γ and IL-17A) by 8 weeks, which limits the growth of established cyst forms in the liver in the murine model. Moreover, the T17-type response also plays complementary roles to the T1-type response in protective immunity against helminth infection (Yasen et al., 2021a). This is consistent with reports from Dematteis, S., who found that early type-2 cytokines were induced by *E. granulosus s.s.* in favor of parasite establishment, since type-2 cytokines are involved in mediating inhibition of macrophage activation (Dematteis et al., 2003). In addition, type-1 cytokines (IFN-γ) may be correlated with the killing of both PSCs and cysts of *E. granulosus s.s.* (Rogan, 1998). Results from CE patients present *ex vivo* evidence that Th1 lymphocytes contribute decisively to the inactive stage of hydatid disease, Th2 lymphocytes in the active and transitional stages (Rigano et al., 2004).

Furthermore, we used CD4 T-cell deficient mice to specifically investigate the role of CD4^+^ T cells in the modulation of the course of *E. granulosus s.s.* infection. Our present data have shown that CD4^+^ T cells appear to play a more crucial role in the immunological control of the development of PSCs into cysts during the establishment phase, in addition to the growth of cysts during the established phage. In recent years, some studies have confirmed that CD4^+^ T cells are involved in controlling parasite load, and their absence leads to significant mortality in helminth infections (Rahemtulla et al., 1991; Dai et al., 2001; Lopez-Briones et al., 2001). Previous studies have shown that immunodeficientmice, such as athymic nude and severe combined immunodeficiency (SCID), exhibited high susceptibility to infection, suggesting that the host immune response plays a major role in suppressing larval growth (Playford and Kamiya, 1992; Playford et al., 1992). Our results are also confirmed by previous data obtained by us and others with regard to murine *E. multilocularis* infections in which mice were treated with CD4-depleting antibodies or subjected to MHC-II deficiency prior to infection (Dai et al., 2004; Zhang et al., 2020).

It is well known that some CD8^+^ T cells are dependent on CD4^+^ T cells to perform their functions during infection (Janssen et al., 2003; Zander et al., 2019; Lu et al., 2021). Several recent findings have shown that CD8^+^ T cells are capable of secreting IL-10 with or without CD4^+^ T-cell help and are involved in the regulation of immune response during virus or parasite infection (Bourreau et al., 2007; Sun et al., 2011; Trandem et al., 2011; Buxbaum, 2015; Jiang et al., 2016). Surprisingly, *E. granulosus s.s.* infection resulted in increased IL-10 expression in hepatic or splenic CD8^+^ T cells from CD4 knockout mice and decreased granzyme B or IFN-γ expression. This suggested that IL-10 contributes to the immune-suppressive environment of lesions in the liver during *E. granulosus s.s.* establishment or during the established phase. Conversely, splenocytes from CD4-depleted mice exhibited a marked elevation in IFN-γ and TNF-α production and reduced production of IL-4, IL-5 and IL-10 compared to those of untreated mice during schistosome infection (Fallon et al., 2000). The results from an *in vivo* implantation experiment showed that there was an increase in IL-10 production and a reduction in IFN-γ production after *E. granulosus* infection. PSC carbohydrates induces the *in vitro* secretion of IL-10 by peritoneal cells derived from normal and infected mice (Dematteis et al., 2001). In addition, a marked increase in suppressor CD8^+^ T cells was observed in the lymph nodes of *E. granulosus*-infected susceptible mice (BALB/c) from days 8-84 p.i. (Riley and Dixon, 1987). In the case of the phylogenetically more related parasite *E. multilocularis*, PSCs are able to induce suppressor CD8^+^ cells in naive splenocyte cultures (Kizaki et al., 1993). Some studies also reported that locally expanded CD8^+^ T cells not only seem to play inefficient roles in helminth infections but are also probably responsible for suppressive immune responses (Little et al., 2005; Metwali et al., 2006).

In summary, our findings showed that T cells were involved in the formation of the immune environment in the liver in CE patients and *E. granulosus s.s.-*infected mice, with CD4^+^ T cells being the dominant immune cells; this process was closely associated with cyst viability and establishment. Moreover, these results demonstrated that during experimental infection by *E. granulosus s.s.*, early and local T2-type immune responses in the liver are permissive for infection establishment between 4 and 6 weeks. Our data, obtained in CD4^+^ T-cell-deficient mice, indicated that CD4^+^ T-cell-mediated cellular immune responses and IL-10-producing CD8^+^ T cells play a crucial role in the establishment phase of secondary *E. granulosus s.s.* PSC infection. This study may help to identify the immunological features of the liver in different developmental stages of CE, including the early establishment and established phases, which in turn could be associated with understanding immunity and designing immunointervention strategies for human secondary infection.

## Data availability statement

The original contributions presented in the study are included in the article/**Supplementary Material**. Further inquiries can be directed to the corresponding authors.

## Ethics statement

The studies involving human participants were reviewed and approved by the ethics committee of First Affiliated Hospital of Xinjiang Medical University (No S20130418-3). Written informed consent to participate in this study was provided by the participants’ legal guardian/next of kin. The animal study was reviewed and approved by the Animal Care and Use Committee and the Ethical Committee of First Affiliated Hospital of Xinjiang Medical University (No 20140411-05).

## Author contributions

Conceived and design: CZ, HW, and JL; performed the experiments: XH, XK, BJ, ZR, AA, MLW, DL, XZ, and YS; analyzed the data: XH and CZ; contributed to materials related issues: LL; drafted the manuscript: CZ, HW, and XH; supervised the study: CZ, JL, and HW; All authors reviewed and approved the final manuscript.

## Funding

The present study was supported financially by National Key Research and Development Program of China (2021YFC2300800, 2021YFC2300801, 2021YFC2300802), National Natural Science Foundation of China (82160396, 81760368, 82060370), Cultivation projects of the National Science Fund for Distinguished Young Scholars (xyd2021J003).

## Acknowledgments

We thank the patients who voluntarily participated in the study.

## Conflict of interest

The authors declare that the research was conducted in the absence of any commercial or financial relationships that could be construed as a potential conflict of interest.

## Publisher’s note

All claims expressed in this article are solely those of the authors and do not necessarily represent those of their affiliated organizations, or those of the publisher, the editors and the reviewers. Any product that may be evaluated in this article, or claim that may be made by its manufacturer, is not guaranteed or endorsed by the publisher.
