# Supplementary material for: Echinococcus granulosus: The establishment of the metacestode in the liver is associated with control of the CD4+ T-cell-mediated immune response in patients with cystic echinococcosis and a mouse model
Source: Front Cell Infect Microbiol. 2022 Aug 15;12:983119. doi: 10.3389/fcimb.2022.983119 (PMC9422084; doi:10.3389/fcimb.2022.983119)

**Supplementary Materials and Methods**

**Liver pathology and fibrosis from CE patients**

Liver tissue samples were fixed in 4% paraformaldehyde (PFA)/PBS for 48 hrs. Dehydrated, paraffin-embedded liver tissue was cut into 5-μm sections, which were then processed for pathological evaluation, Masson’s trichrome staining and immunohistochemistry. The sections were examined microscopically for specific staining and photographs were taken using a digital image-capture system (Olympus, Tokyo).

For pathological analysis, sections were stained with hematoxylin and eosin (H&E) to evaluate infiltrating cells and liver immune microenvironment formation according to guidelines for clinical samples. The tissue sections were deparaffinized with xylene twice, hydrated with alcohol, and stained with hematoxylin for 3 min. Afterward, sections were treated with 1% hydrochloric acid alcohol, stained with eosin for 5 min, dehydrated, transparentized with xylene, and mounted with neutral resin, followed by observation of liver pathological changes under the microscope (Yang et al., 2022).

For fibrosis analysis, liver sections from CE patients were stained by Masson’s staining (Polysciences, Warrington, PA) according to the manufacturer’s standard protocols. In brief, sections were stained with Masson for 5 min, with ponceau- magenta for 5 min, with phosphomolybdic acid for 2 min, and with aniline blue for another 2 min. The sections were subsequently treated within the differentiation solution, hydrated with alcohol, transparentized with xylene, mounted with neural resin, and observed under a microscope for liver fibrosis level (Yang et al., 2022).

**Tissue sampling and histopathological analysis from mice model**

For liver histopathology, all liver lobes were separated and placed in 10% buffered formalin and then embedded in paraffin. Paraffin-embedded 5 μm sections were mounted on glass slides and stained with the H&E to evaluate infiltrating cells and granuloma formation. Liver histological reactions surrounding the infectious foci were classified into three categories (a) ‘PSCs foci’, visible PSCs, only composed of infiltrating inflammatory cells and fibrosis; (b) ‘infectious foci or hydatid cyst’, cystic structure composed of the germinal layer and laminated layer, surrounded with macrophages, lymphocytes, fibroblasts, myofibroblasts, as well as fibrosis (adventitial layer); (c) ‘inflammatory foci', parasite-free, except for possible PSC remnants, composed of macrophages, lymphocytes, and other inflammatory cells. This result was expressed as the number of each type of infectious focus by observation under the microscope.. The lesion areas in 5–10 fields/section/mouse (× 100) were measured by computer-assisted morphometric analysis using cellSens Dimension software (Olympus, Tokyo, Japan) and expressed as square micrometers (mm^2^).

For liver fibrosis, paraffin-embedded 5 μm sections were stained with the picric acid-Sirius red technique to evaluate collagen fibers, as described previously (Heinrichs et al., 2013). After heating at 60 °C for 2 h, sections were deparaffinized, immersed in distilled water for 2 min, stained with Harris hematoxylin solution for 3–5 min, and washed with distilled water thrice. Later, sections were stained with Sirius Red staining solution at 37°C for 25 min, color-separated and dehydrated with absolute ethyl alcohol, transparentized with xylene, dried in the air, and mounted with neutral resin. This was assessed at × 200 magnifications in a total of 3–5 fields/section/ sample, using computerized quantification and results were expressed as percentage of picric acid-sirius red staining per field.

Reference

Heinrichs, D., Berres, M.L., Nellen, A., Fischer, P., Scholten, D., Trautwein, C., Wasmuth, H.E., and Sahin, H. (2013). The chemokine CCL3 promotes experimental liver fibrosis in mice. PloS one *8*, e66106.

Yang, Y., Sheng, Y., Wang, J., Zhou, X., Li, W., Zhang, C., Guo, L., and Han, C. (2022). Double-Negative T Cells Regulate Hepatic Stellate Cell Activation to Promote Liver Fibrosis Progression via NLRP3. Frontiers in immunology *13*, 857116.

**Table S1 Baseline clinical characteristics of CE patients studied**

| **Patient No.** | **Age(years)** | **Gender（F/M）** | **Stages of CE** | **ALT (U/L)** | **AST (U/L)** | **ALP (U/L)** | **Characterization of cysts [location, diameter (cm)]** | **Whether or recurrence** | **Surgical approach** |
| --- | --- | --- | --- | --- | --- | --- | --- | --- | --- |
| 1 | 13 | F | 1 | 172.0 | 124.0 | 61.0 | RL, 8.0×7.0; RL, 2.0×2.0 | N | TC |
| 2 | 42 | F | 1 | 1.1 | 10.0 | 43.0 | LL, 3.3×3.5 | Y | TC |
| 3 | 47 | M | 1 | 95.0 | 37.0 | 101.0 | RL, 8.0×7.0 | Y | TC |
| 4 | 85 | M | 1 | 41.0 | 20.0 | 136.0 | RL, 10.0×11.0 | N | SC |
| 5 | 48 | F | 1 | 103.0 | 38.0 | 66.0 | RL, 6.0×6.0; RL, 3.0×2.0 | Y | TC |
| 6 | 55 | F | 1 | 86.0 | 111.0 | 78.0 | RL, 13.4×10.3, RL, 4.0×4.0 | N | TC, SC, HT |
| 7 | 18 | M | 1 | 23.1 | 18.6 | 45.8 | RL, 5.0×6.0 | N | TC |
| 8 | 60 | M | 1 | 20.3 | 13.7 | 82.5 | RL, 3.0×7.0; 4.0×6.0 | N | HD, SC |
| 9 | 66 | F | 1 | 105.3 | 92.4 | 61.3 | RL, 10.0×8.0; 6.0×5.0 | N | TC |
| 10 | 36 | M | 1 | 160.3 | 79.3 | 187.0 | RL, 12.0×10.0; ML, 10.0×8.0 | N | TC |
| 11 | 54 | F | 1 | 24.0 | 22.3 | 73.6 | RL, 10.0×6.0 | Y | TC |
| 12 | 14 | F | 1 | 17.8 | 20.3 | 124.0 | RL, 11.0×7.0 | Y | TC |
| 13 | 45 | M | 1 | 114.7 | 102.9 | 127.2 | RL, 7.0×10.0; 6.0×7.0 | Y | TC |
| 14 | 44 | F | 1 | 55.8 | 37.1 | 167.0 | RL, 10.1×7.8 | N | TC |
| 15 | 38 | M | 1 | 140.3 | 112.0 | 39.7 | LL, 12.0×10.0 | N | TC |
| 16 | 29 | F | 1 | 9.2 | 15.3 | 95.1 | LL, 5.0×7.0 | N | TC |
| 17 | 76 | F | 1 | 71.0 | 73.5 | 103.7 | LL, 9.0×8.0 | N | HT |
| 18 | 50 | M | 1 | 39.9 | 29.0 | 71.7 | RL, 7.0×6.0 | N | HT |
| 19 | 59 | F | 1 | 18.5 | 18.1 | 75.0 | RL, 9.0×7.0; RL, 2.0×1.0 | N | SC, HD, TC |
| 20 | 36 | F | 1 | 18.1 | 19.7 | 68.0 | LL, 8.8×9.0 | N | SC, HD |
| 21 | 48 | F | 2 | 29.3 | 22.3 | 129.4 | RL, 13.5×17.6; RL, 14.6×6.7 | N | TC, SC, HD |
| 22 | 25 | F | 2 | 10.0 | 11.7 | 49.0 | RL, 6.0×7.0; RL, 3.0×3.0; LL, 2.0×2.0 | N | TC, SC, HD |
| 23 | 38 | M | 2 | 53.5 | 43.4 | 210.1 | LL, 9.0×6.0 | N | TC |
| 24 | 17 | M | 2 | 1.1 | 48.1 | 80.0 | RL, 17.0×16.0 | Y | HD |
| 25 | 25 | M | 2 | 61.6 | 40.1 | 97.1 | LL, 13.0×10.0 | N | TC |
| 26 | 44 | F | 2 | 137.0 | 143.0 | 44.0 | RL, 12.0×10.0; RL, 6.0×5.0 | Y | TC |
| 27 | 25 | F | 2 | 26.0 | 19.0 | 76.0 | RL, 8.0×6.0; RL, 6.0×4.0 | N | TC |
| 28 | 32 | M | 2 | 16.0 | 15.6 | 69.0 | RL, 9.0×7.0 | N | TC |
| 29 | 31 | F | 2 | 33.0 | 48.0 | 111.0 | LL, 8.0×3.0 | N | TC |
| 30 | 25 | M | 2 | 14.9 | 19.3 | 102.8 | RL, 8.0×7.0 | Y | TC |
| 31 | 19 | F | 2 | 11.9 | 20.1 | 111.6 | RL, 12.0×10.0; 10.0×8.0; 6.0×5.0 | N | TC |
| 32 | 43 | M | 2 | 107.3 | 57.1 | 47.0 | LL, 8.0×8.0 | Y | TC, HT |
| 33 | 61 | F | 2 | 19.6 | 18.4 | 63.0 | LL, 6.0×6.0 | N | TC |
| 34 | 22 | F | 2 | 20.0 | 18.7 | 84.0 | RL, 9.0×6.0; 6.0×5.0 | N | TC, HT |
| 35 | 29 | F | 2 | 14.9 | 18.3 | 77.0 | ML, 8.0×8.0 | N | TC, HT |
| 36 | 50 | F | 2 | 22.4 | 27.9 | 89.0 | RL, 12.0×10.0; 6.0×5.0 | Y | TC |
| 37 | 26 | M | 2 | 15.6 | 20.4 | 98.0 | RL, 5.0×3.0 | N | HD |
| 38 | 50 | F | 2 | 51.7 | 107.8 | 754.0 | RL, 18.0×11.0 | N | TC |
| 39 | 30 | F | 2 | 304.3 | 224.7 | 48.6 | LL, 4.0×4.3; 5.0×6.0 | N | TC |
| 40 | 36 | M | 2 | 17.1 | 13.0 | 75.8 | RL, 6.0×5.5 | N | TC |
| 41 | 43 | F | 2 | 166.9 | 45.3 | 91.0 | RL, 16.0×12.0 | Y | TC |
| 42 | 40 | F | 2 | 29.4 | 25.8 | 39.1 | RL, 18.0×15.0 | N | SC, HD |
| 43 | 58 | M | 2 | 38.7 | 32.4 | 85.0 | ML, 15.0×14.0 | N | TC |
| 44 | 20 | M | 2 | 7.7 | 13.0 | 77.0 | LL, 11.0×10.0 | N | TC |
| 45 | 37 | F | 2 | 29.7 | 26.7 | 64.4 | RL, 13.0×12.0 | N | SC, HD |
| 46 | 32 | M | 2 | 105.5 | 46.8 | 241.2 | LL, 9.0×10.0 | N | SC, HD |
| 47 | 40 | M | 2 | 163.0 | 82.1 | 102.2 | RL, 6.0×5.0 | N | TC |
| 48 | 28 | M | 2 | 36.3 | 22.7 | 101.0 | RL, 9.0×10.0 | Y | TC |
| 49 | 32 | M | 2 | 63.5 | 32.2 | 132.0 | RL, 9.0×7.0; LL, 5.0×6.0 | N | HT |
| 50 | 20 | F | 2 | 19.7 | 22.2 | 116.1 | RL, 12.0×8.0 | N | HT |
| 51 | 20 | F | 2 | 59.0 | 15.2 | 116.0 | RL, 12.0×8.0; 8.0×7.0 | N | HT |
| 52 | 28 | F | 2 | 27.1 | 29.4 | 78.4 | LL, 10.0×9.0 | N | TC |
| 53 | 54 | M | 2 | 11.5 | 119.6 | 208.3 | RL, 13.0×10.0 | N | HT |
| 54 | 45 | F | 2 | 13.8 | 18.1 | 75.0 | RL, 9.0×7.0 | N | TC |
| 55 | 59 | M | 2 | 147.7 | 70.9 | 55.5 | RL, 9.0×7.0 | N | HT |
| 56 | 68 | F | 2 | 195.0 | 128.4 | 80.9 | LL, 9.0×7.0 | N | HT |
| 57 | 20 | F | 2 | 9.0 | 19.6 | 85.5 | RL, 9.0×7.0 | N | TC |
| 58 | 52 | F | 2 | 16.3 | 14.6 | 97.0 | LL, 9.0×7.0 | N | TC |
| 59 | 50 | M | 2 | 183.3 | 62.3 | 97.8 | RL, 12.0×7.0 | N | TC |
| 60 | 45 | M | 2 | 249.9 | 115.0 | 70.3 | LL, 8.0×8.0 | N | TC |
| 61 | 56 | M | 2 | 103.2 | 171.7 | 106.1 | RL, 12.0×10.0 | N | TC |
| 62 | 60 | F | 2 | 126.0 | 88.4 | 62.9 | RL, 5.0×4.0; LL, 9.0×7.0 | N | HD, SC |
| 63 | 44 | M | 2 | 12.9 | 17.1 | 69.5 | LL, 9.0×8.0 | N | HT |
| 64 | 50 | F | 2 | 26.4 | 19.3 | 83.0 | RL, 9.0×7.0 | N | TC |
| 65 | 44 | M | 2 | 126.9 | 74.7 | 160.4 | RL, 9.2×8.3 | N | SC, HD |
| 66 | 49 | F | 2 | 15.7 | 12.0 | 83.6 | RL, 7.5×8; LL, 3.1×2.8 | N | TC, SC, HD |
| 67 | 21 | M | 2 | 13.5 | 14.3 | 99.0 | RL, 5.5×4.8 | N | TC |
| 68 | 41 | M | 2 | 112.6 | 80.2 | 344.9 | LL, 4.3×3.3 | N | HT |
| 69 | 37 | F | 2 | 13.5 | 16.3 | 63.0 | LL, 5.8×5.5 | N | HT |
| 70 | 36 | M | 2 | 17.1 | 14.3 | 56.1 | RL, 5.6×4.6 | N | TC |
| 71 | 45 | M | 2 | 19.0 | 12.0 | 85.0 | RL, 8.9×9.3 | Y | SC, HD |
| 72 | 47 | F | 2 | 13.0 | 13.8 | 62.0 | RL, 6.5×5.1 | N | SC, HD |
| 73 | 43 | M | 2 | 31.8 | 24.6 | 130.0 | RL, 14.8×11.5 | N | SC, HD |
| 74 | 55 | F | 2 | 16.9 | 18.3 | 72.0 | RL, 7.9×7.0 | N | TC |
| 75 | 50 | F | 2 | 11.4 | 13.9 | 78.0 | RL, 12.3×9.4 | N | SC, HD |
| 76 | 34 | M | 2 | 40.0 | 28.6 | 105.5 | LL, 9.5×6.7 | N | SC, HD |
| 77 | 53 | M | 2 | 21.5 | 27.1 | 86.0 | RL, 8.9×7.7 | Y | SC, HD |
| 78 | 20 | M | 2 | 14.2 | 14.3 | 100.0 | ML, 8.5×6.3 | N | HT |
| 79 | 48 | F | 3 | 19.0 | 17.0 | 94.7 | RL, 13.5×13.4 | N | HT |
| 80 | 49 | M | 3 | 14.5 | 14.0 | 90.0 | LL, 12.7×7.9 | N | HT |
| 81 | 23 | F | 3 | 55.0 | 37.0 | 56.0 | LL, 5.0×6.0 | Y | TC |
| 82 | 32 | M | 3 | 59.0 | 26.0 | 84.0 | RL, 6.1×5.5 | N | TC |
| 83 | 39 | M | 3 | 10.6 | 23.3 | 90.6 | ML, 8.0×8.0 | N | TC |
| 84 | 35 | F | 3 | 16.3 | 22.4 | 45.2 | RL, 8.3×4.5 | Y | TC |
| 85 | 52 | F | 3 | 13.0 | 19.1 | 126.0 | RL, 3.0×5.0; 7.0×6.0 | N | TC |
| 86 | 29 | M | 3 | 24.8 | 15.3 | 54.5 | RL, 8.0×6.0 | N | TC |
| 87 | 17 | M | 3 | 42.8 | 55.9 | 44.3 | RL, 15.0×15.0 | N | TC |
| 88 | 52 | F | 4 | 9.7 | 19.0 | 105.0 | RL, 9.0×9.0 | N | TC |
| 89 | 46 | M | 4 | 21.6 | 19.7 | 46.9 | RL, 8.5×6.8 | N | SC, HD |
| 90 | 42 | M | 4 | 350.7 | 268.5 | 454.7 | LL, 5.9×4.4 | Y | HT |
| 91 | 42 | M | 4 | 16.0 | 20.0 | 94.0 | RL, 4.5×6.4 | N | TC |
| 92 | 48 | F | 4 | 18.7 | 22.6 | 82.4 | RL, 6.0×6.0 | N | TC, SC, HT |
| 93 | 12 | F | 4 | 56.0 | 65.0 | 203.0 | LL, 7.0×6.0 | N | TC |
| 94 | 43 | M | 4 | 181.4 | 139.8 | 53.0 | RL, 10.0×8.0; LL, 6.0×5.0 | Y | TC, SC, HT |
| 95 | 55 | M | 4 | 142.8 | 110.5 | 62.6 | LL, 5.0×4.0; LL, 4.0×3.0 | N | TC, HC |
| 96 | 47 | M | 4 | 99.0 | 78.0 | 50.0 | LL, 6.0×5.0 | N | TC, HT |
| 97 | 21 | F | 4 | 33.5 | 30.7 | 69.4 | RL, 6.0×5.0 | N | TC |
| 98 | 33 | M | 4 | 62.4 | 26.4 | 119.0 | RL, 10.0×8.0 | N | TC, HT |
| 99 | 33 | M | 4 | 20.7 | 17.8 | 59.2 | RL, 8.0×8.0 | N | TC |
| 100 | 68 | F | 4 | 12.2 | 17.5 | 100.0 | RL, 7.0×6.0 | N | TC |
| 101 | 60 | M | 4 | 43.6 | 23.6 | 79.0 | LL, 18.0×15.0 | N | HT |
| 102 | 42 | M | 4 | 45.0 | 25.8 | 75.8 | RL, 16.9×13.6 | N | TC |
| 103 | 25 | F | 4 | 41.4 | 26.9 | 66.6 | RL, 8.0×8.0 | N | TC |
| 104 | 40 | M | 4 | 50.4 | 49.4 | 65.3 | LL, 12.0×10.0 | N | TC |
| 105 | 27 | M | 4 | 59.5 | 143.6 | 65.0 | RL, 8.0×8.0 | N | TC |
| 106 | 46 | M | 4 | 145.5 | 126.8 | 36.7 | LL, 8.0×6.0 | N | TC |
| 107 | 52 | M | 4 | 31.0 | 19.1 | 83.0 | RL, 16.2×15.9 | N | TC |

Abbreviations: F, female; M, male; LL, left liver lobe; RL, right liver lobe; ML, middle liver lobe; ALT, alanine aminotransferase; AST, aspartate aminotransferase; ALP, alkaline phosphatase; +, positive; ―, negative; TC: Total cystectomy; HT: Hepatectomy; SC: Subtotal cystectomy; HD: Hydatidectomy (endocystectomy); N, none; Y, yes; NA, not available.

**sFigure 1. Assessment of the levels of serum ALT, AST and ALP in CE patients at different stages**

Data are expressed as mean ± SD, analyzed by Student’s t tests or one way ANOVA. **P* < 0.05, n.s., *P* > 0.05.

**sFigure 2. Fibrosis assessment and a-Sma staining in the liver in mouse model during *E. granulosus s.s.* establishment**

(A) Liver fibrosis as determined by picric acid-Sirius red staining (original magnification 200×) from mice model during *E. granulosus s.s.* establishment. The red area represents fibrillar collagen. (B) Representative immunohistochemistry staining for α-Sma on liver sections (original magnification 100×) from mice model during *E. granulosus s.s.* establishment. (C) The fibrosis area of the section was quantified using cellSens Dimension software, the ratio of collagen area and total area (%) was counted, from (A). (D) The percentage of positive staining cells was calculated to assess the expression of α-Sma on the lesion areas, from (B). 4-5 mice per group. PSCs: protoscoleces. Cyst: hydatid cyst. The dotted box represents the absence of PSC-type lesions. All data are presented as mean.

**sFigure 3. Fibrosis assessment of the liver in WT or CD4 T-cell-deficient mice during *E. granulosus s.s.* establishment**

(A) Representative liver fibrosis as determined by picric acid-Sirius red staining (left panel 40×, enlarged 100× on the right panel) from WT or CD4 KO mice at week 10 post infection. (B) Representative immunohistochemistry staining for α-Sma (left panel 40×, enlarged 100× on the right panel) from WT or CD4 KO mice at week 10 post infection. (C) The positive staining area of the section was quantified using cellSens Dimension software. 4-5 mice per group. All data are presented as mean ± SD. Data was analyzed using Student’s t tests. n.s., *P* > 0.05.

**sFigure 4. CD8^+^ T cells expressing granzyme B, IFN-γ and IL-10 in the spleen in wild-type or CD4 KO mice after *E. granulosus s.s.* infection**

(A) Representative flow cytometry plot of granzyme B, IFN-γ and IL-10 production in CD8^+^ T cells in the spleen from WT or CD4 KO mice at week 10 post infection. (B) The percentage of granzyme B, IFN-γ and IL-10 production in CD8^+^ T cells in the liver, from (A). 4-5 mice per group. All data are presented as mean ± SD. Data was analyzed using Student’s t tests. ****P* < 0.001, n.s., *P* > 0.05.

**sFigure 1. Assessment of the levels of serum ALT, AST and ALP in CE patients at different stages**


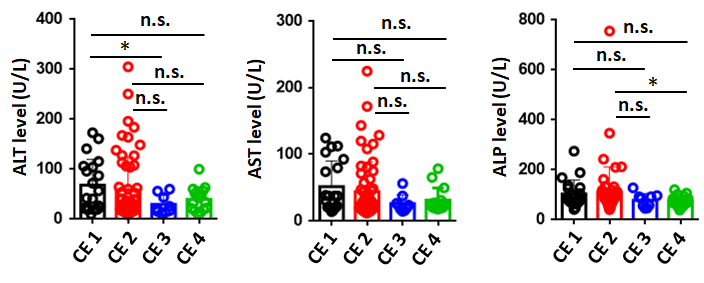


**sFigure 2. Fibrosis assessment and a-Sma staining in the liver in mouse model during *E. granulosus s.s.* establishment**


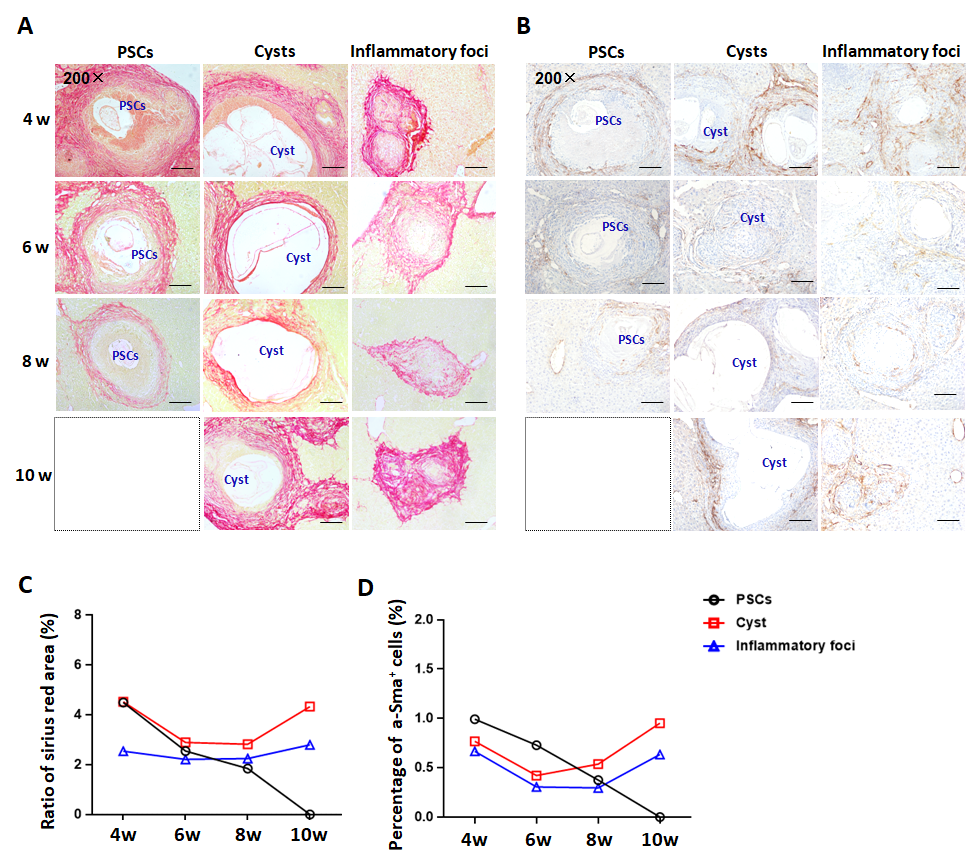
 **sFigure 3. Fibrosis assessment of the liver in WT or CD4 T-cell-deficient mice during *E. granulosus s.s.* establishment**


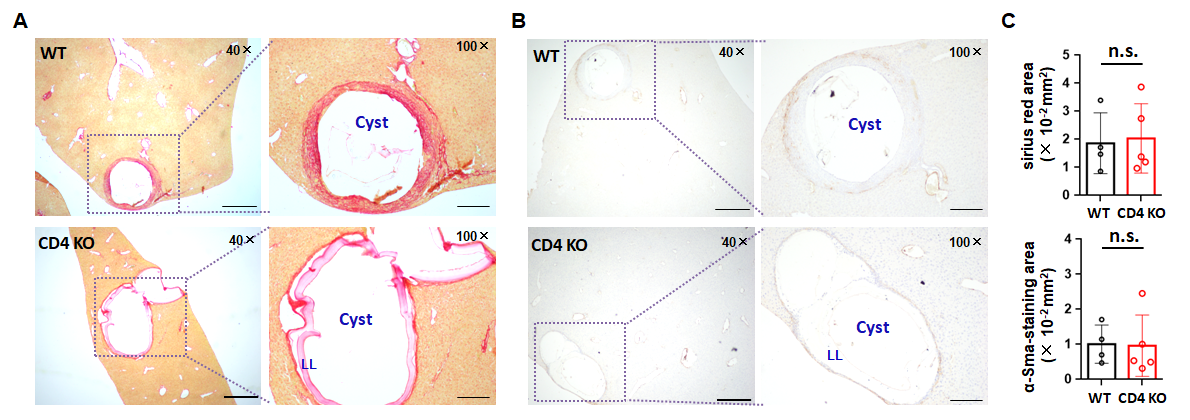
 **sFigure 4. CD8^+^ T cells expressing granzyme B, IFN-γ and IL-10 in the spleen in wild-type or CD4 KO mice after *E. granulosus s.s.* infection**


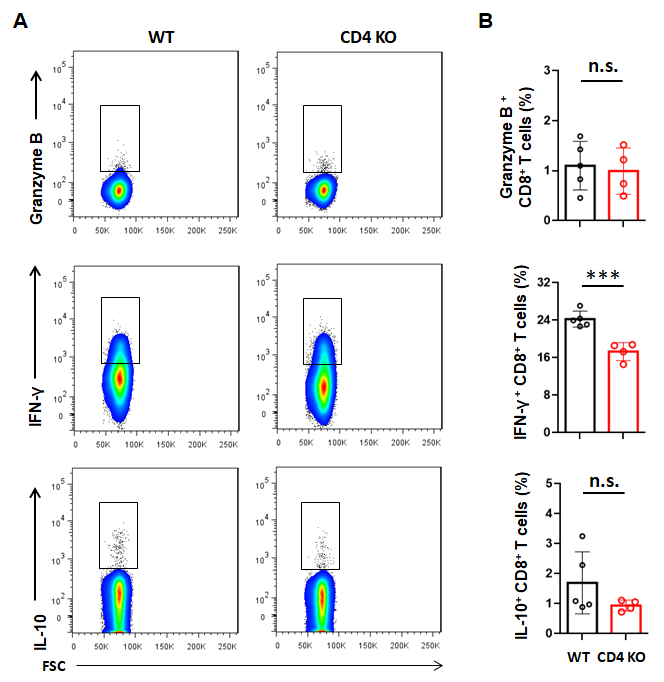

Supplement: Supplementary file 1 [file DataSheet_1.docx]
